# Organizational resilience and internal branding: investigating the effects triggered by self-service technology

**DOI:** 10.1057/s41262-022-00275-9

**Published:** 2022-04-07

**Authors:** Galina Biedenbach, Thomas Biedenbach, Peter Hultén, Veronika Tarnovskaya

**Affiliations:** 1 Umeå School of Business, Economics and Statistics, Umeå University, Biblioteksgränd 6, 901 87 Umeå, Sweden; 2 Dalarna University, Röda vägen 3, 781 70 Borlänge, Sweden; 3 Lund School of Economics and Management, Lund University, Tycho Brahes väg 1, Box 7080, 220 07 Lund, Sweden

**Keywords:** Internal brand equity, Internal branding, Resilience, Retailing, Self-service, SST

## Abstract

The majority of studies on internal brand equity examine its various dimensions and relationships between them. While prior research specifies organizational practices relevant for successful internal branding, the insights about the impact of essential organizational factors on internal brand equity are still limited. This study focuses on organizational resilience that is vital for the existence of organizations not only during a crisis, but also during everyday operations. The main purpose of this study is to investigate the impact of organizational resilience on internal brand equity considering the effects triggered by self-service technology (SST) in retailing. Since retailing had been significantly transformed by technological innovations over the past decade, we explore the effects of employees’ perceptions about performance of SST. The results of a survey conducted among retail employees in Sweden demonstrate that organizational resilience and employees’ perceptions about technological innovations are critical for enhancing internal brand equity, which includes brand orientation, internal brand knowledge, internal brand involvement, and internal brand commitment.

## Introduction

In 2020, the unexpected spread of a novel coronavirus SARS-CoV-2 across the world resulted in the COVID-19 pandemic that caused a shocking loss of more than five million of human lives over 2 years and made an unprecedented devastating impact on businesses and other organizations. In this context, to withstand the hardships, resilience is undoubtedly a critical capability determining the survival of individuals, companies, and the entire society. Psychology and organization studies confirm that resilience is of the utmost importance for dealing with unexpected challenges requiring adaptability, flexibility, and bouncing back both by individuals and by organizations (Youssef and Luthans 2007; Fletcher and Sarkar 2013; Britt et al. 2016; Linnenluecke 2017; Liu et al. 2019; Tarba et al. 2019). Organizational resilience represents a capability that enables organizations “to effectively absorb, develop situation-specific responses to, and ultimately engage in transformative activities to capitalize on disruptive surprises that potentially threaten organization survival” (Lengnick-Hall et al. 2011, p. 244). Furthermore, prior research brings attention to the fact that resilience is relevant not only for contexts involving significant adversity and extreme environments, but also for everyday existence and stable environments (Kuntz et al. 2016; Branicki et al. 2019; Stokes et al. 2019). In this study, we answer to the calls for research exploring organizational resilience in the workplace and examining the micro-processes critical for developing a resilient organization during day-to-day operations (Branicki et al. 2019; Hartmann et al. 2020).

Although resilience certainly represents a phenomenon of high importance for organizations, the notion of a resilient organization and organizational resilience are not well explored in branding research. The majority of branding studies considering resilience apply a consumer perspective and provide empirical evidence about consumer resilience to negative information (Elbedweihy et al. 2016; Japutra et al. 2018; Torres and Augusto 2019), resilience intentions (Jung et al. 2021), and resilience of brand attitudes (Sweldens et al. 2010). By applying another theoretical perspective, previous studies on place branding investigate resilience in relation to city branding (Coaffee and Rogers 2008; Naef 2020) and nation branding (Tamaki 2019). In addition, very few works in the branding literature examine the relevance of brand resilience for organizational success (e.g. Testa et al. 2017; Boukis et al. 2021). In general, prior branding research provides scattered theoretical propositions and limited empirical evidence about resilience. In this study, we address these research gaps by applying an employee perspective, through which we examine the impact of organizational resilience on the key outcome of internal branding that is internal brand equity.

We conceptualize internal brand equity as “the incremental effect of branding on employee behaviour” (Baumgarth and Schmidt 2010, p. 1250). Following a contemporary perspective on internal branding, each employee in an organization can be seen as “an active force and decision maker in internal branding, and thus brand cocreation” (Merrilees et al. 2021, p. 813). Therefore, each employee’s perceptions of, and responses to, internal branding are central for building a strong organizational brand. Based on the propositions from previous studies, we examine the four core dimensions of internal brand equity, which are brand orientation, internal brand knowledge, internal brand involvement, and internal brand commitment (Baumgarth and Schmidt 2010). While prior branding research provides insights about the main mechanisms of internal branding and relationships between the dimensions of internal brand equity, there is still a lack of knowledge about the organizational factors affecting an internal brand and determining success of internal branding (Saleem and Iglesias 2016; Iyer et al. 2018; Piehler et al. 2018; Leijerholt et al. 2019; Boukis and Christodoulides 2020; Barros-Arrieta and García-Cali 2021). Drawing on previous resilience studies, which highlight the criticality of organizational resilience in shaping employees’ attitudes and behaviours in a workplace (Kuntz et al. 2016; Hartmann et al. 2020; Annarelli et al. 2020), this study advances the current state of knowledge on internal branding by assessing how organizational resilience influences the dimensions of internal brand equity.

In a workplace, organizational resilience is reinforced or, in a negative case, diminished during day-to-day operations. In this everyday resilience setting, we contend that an overlooked area in extant research is how technological innovations impact employees’ perceptions and ultimately these innovations influence internal brand equity. In retailing, successful implementation of self-service technology (SST), such as self-scanning devices, self-payment machines, and mobile applications, can increase the operational efficiency for retailers (Sorescu et al. 2011), facilitate shopping for consumers (Meuter et al. 2000), and even rise the effectiveness for the entire ecosystem of actors functioning along customer journeys (Singh et al. 2019). However, by transforming the roles of consumers and employees during service encounters, technological innovations can also lead to negative consequences (Larivière et al. 2017). For example, some consumers can feel self-conscious (Dabholkar and Bagozzi 2002), anxious (Meuter et al. 2003), incompetent (Bulmer et al. 2018), or confused (Johnson et al. 2021) when using SST. Despite such advantages of SST as the speed of service and job performance, employees can experience an increased workload, additional stress due to ambiguous tasks, as well as concerns about their future retention (Hsieh and Yen 2005; Di Pietro et al. 2014). Negative perceptions of employees about SST can result in lower morale and decreased satisfaction, which would consequently have negative effects on their interactions with consumers and commitment to their employer (Verhoef et al. 2009). Even though previous studies on SST indicate a strong impact of SST on attitudes and behaviours of employees, they predominantly focus on consumers and provide disproportionally limited empirical evidence about employee-related and firm-centric outcomes (Di Pietro et al. 2014; Taillon and Huhmann 2019).

The main purpose of this study is to investigate the impact of organizational resilience on internal brand equity considering the effects triggered by SST in retailing. As a research context, we selected grocery stores in Sweden and focused on retail employees’ perceptions of their day-to-day operations. This choice was driven by the fact that digital transformation in retailing resulted in the emergence of new technological innovations, which must be seized not only by large chains, but also by small stores aiming to achieve success in a competitive marketplace. Therefore, SST is increasingly used by retailers to stay at the forefront of technological progress. Considering that SST adds complexity to day-to-day operations in grocery stores and can potentially mediate the effects between organizational resilience and internal brand equity in these workplaces, we conducted an online survey by involving a panel of employees working at grocery stores, which had implemented SST. We initiated our study prior to the pandemic and explored the role of organizational resilience in everyday work environments and its impact on employees’ perceptions of their organizational brands. Later during the research process, we were surprised and shocked to see how an unexpected pandemic had rapidly changed grocery stores to contagious environments and how their employees became frontline essential workers, who practically demonstrated that organizational resilience represents a vital capability for organizations, which traditionally were not considered as extreme environments. The study makes a theoretical contribution to research on internal branding by examining employees’ perceptions about organizational resilience, SST, and internal brand equity. Furthermore, we provide practical recommendations for managers on how to enhance internal brand equity by proactively strengthening organizational resilience and mitigating the adverse effects triggered by technological innovations on their employees and organizational brands.

## Theoretical framework

### Internal brand equity

The stream of research on internal branding evolved from classical assumptions stressing the vital importance of committed employees for the effective functioning and performance of an organization (Sasser and Arbeit 1976; Berry 1981). According to the contemporary view, internal branding incorporates organizational efforts devoted “to enable employees to consistently co-create brand value with multiple stakeholders” (Saleem and Iglesias 2016, p. 50). The findings of previous studies demonstrate that successful internal branding can positively influence attitudes of employees towards an organizational brand, as well as affect their behavioural intentions (Burmann and Zeplin 2005; King and Grace 2008; Foster et al. 2010; Boukis and Christodoulides 2020; Carlini and Grace 2021; Leijerholt 2021). Consequently, a strong internal brand can support the implementation of corporate programmes (Ahmed and Rafiq 2003), increase the quality of brand–consumer relationships (Burmann et al. 2009), improve financial performance (Tuominen et al. 2016), and facilitate the development of competitive advantage (Gapp and Merrilees 2006), among other benefits.

The literature on internal branding highlights that internal brand equity, which is also referred to as employee-based brand equity or employee brand equity, represents the ultimate goal of internal brand management (King and Grace 2010; King et al. 2012; Schmidt and Baumgarth 2018; Jacobson et al. 2021). In general, internal brand equity captures “the strength of workforce internalization of brand identity, in support of branding at the customer interface” (Baumgarth and Schmidt (2010, p. 1250). Previous studies on internal branding provide a number of alternative models specifying the dimensions of internal brand equity (e.g. Baumgarth and Schmidt 2010; Piehler et al. 2016; Boukis and Christodoulides 2020). Considering the propositions made in prior research, we focus on the four core dimensions of internal brand equity, which are brand orientation, internal brand knowledge, internal brand involvement, and internal brand commitment (Baumgarth and Schmidt 2010). First, brand orientation originating from a strategic perspective on internal brand management (Urde 1994, 1999) is conceptualized as “a specific type of strategic orientation or corporate culture, characterized by high relevance of the brand as the basis of the business model” (Baumgarth and Schmidt 2010, p. 1252). Second, capturing the cognitive responses of employees, internal brand knowledge represents “brand-related cognitive schemata” (Boukis and Christodoulides 2020, p. 45). Third, centred around the affective responses of employees, internal brand involvement embraces “the personal relevance of the brand” (Baumgarth and Schmidt 2010, p. 1253). Finally, internal brand commitment characterizes “an employee's psychological attachment to the brand” (Baumgarth and Schmidt 2010, p. 1253).

While earlier studies provide important insights about internal brand equity, there is still a lack of consensus about the specific relationships established between its core dimensions. Nevertheless, the literature on internal branding provides empirical evidence indicating a possible hierarchy of effects evolving from brand orientation to internal brand knowledge, and then to internal brand involvement, and consequently to internal brand commitment. For example, a study involving employees and managers of business-to-business companies demonstrates a positive effect of brand orientation on internal brand knowledge (Baumgarth and Schmidt 2010). A study conducted among service employees shows the positive impact of cognitive outcomes of internal brand management (e.g. brand understanding including brand knowledge) on affective outcomes (e.g. brand identification underlying internal brand involvement) (Piehler et al. 2016). In addition, another study conducted among employees of service organizations emphasizes the relevance of assessing cognitive and affective responses to an organizational brand for enhancing internal brand equity (Boukis and Christodoulides 2020). A study involving frontline employees in the service industry confirms the positive impact of cognitive brand understanding, such as employee brand knowledge, on affective brand connection, such as employee brand identification (Ngo et al. 2020). Furthermore, prior research supports the assumptions about the impact of internal brand involvement on internal brand commitment (Piehler et al. 2016). Considering these findings, we propose the hypotheses about the potential hierarchical effects between the four dimensions of internal brand equity.

#### H1a

Brand orientation has a positive effect on internal brand knowledge.

#### H1b

Internal brand knowledge has a positive effect on internal brand involvement.

#### H1c

Internal brand involvement has a positive effect on internal brand commitment.

### Organizational resilience

Theoretical developments in psychology research have resulted in the evolution of the resilience concept from being considered as “an extraordinary, special gift that only a few people possessed” to “the positive psychological capacity to rebound, to ‘bounce back’ from adversity, uncertainty, conflict, failure or even positive change, progress and increased responsibility” (Luthans 2002, p. 702). Earlier studies on resilience emphasize the importance of individual resilience, which represents a high degree of “dynamic resourcefulness in maintaining a personally sufficient adaptational system” (Block and Kremen 1996, p. 351). Advancing this perspective on resilience, later studies on resilience highlight the relevance of organizational resilience, which captures the ability of an organization “to respond appropriately to unexpected situations” (Mu and Butler 2009, p. 32). A bibliographic mapping of business and management research on resilience demonstrates that the initial research streams investigating organizational responses to adversity, organizational reliability, and employee strengths have been complemented by additional research streams exploring the general adaptability of business models and even resilient designs of supply chains (Linnenluecke 2017). The resilience literature proposes that organizations need to develop strategies to ensure a continuous enhancement of resilience, and to implement routines for mitigating conditions, which would hinder it (Hartmann et al. 2020). Furthermore, prior research emphasizes the critical role of employees in increasing organizational resilience (Kahn et al. 2018; Branicki et al. 2019).

In organization studies, resilience at the individual, team, and organizational levels has been widely acknowledged as an important factor influencing attitudinal and behavioural outcomes in the workplace (Youssef and Luthans 2007; King et al. 2016). While prior branding research demonstrates the relevance of considering employees’ personal characteristics (e.g. age, education, length of service) for successfully implementing internal branding (Punjaisri and Wilson 2011), the impact of resilience is still relatively unexplored. Nevertheless, organizational psychology research indicates that a positive adaptation resulting from applying employee resilience capacity leads to positive outcomes, for example, such as increased job performance, high levels of well-being, and healthy relationships (Britt et al. 2016). Prior research suggests that resilience building initiatives implemented by an organization have the potential to foster resilient employee behaviour, as well as facilitate learning, collaboration, and engagement in the entire organization (Kuntz et al. 2017). As a result, the efforts devoted to strengthening organizational resilience can in turn have positive effects on employees’ understanding of brand identity, brand relevance, and brand values, which form brand orientation. Prior research highlights that the development of organizational resilience is characterized by the emergence and use of cognitive, behavioural, emotional, and relational capability endowments, which evolve over time (Williams et al. 2017). These various resource endowments are likely to influence positively individuals within an organization and their perceptions about it (Williams et al. 2017). Previous studies confirm that organizational resilience capacity can be expected to influence positively not only general firm performance, but also other diverse desirable outcomes related to employee contributions and embracing various cognitive, behavioural, and contextual dimensions (Lengnick-Hall et al. 2011). Therefore, organizational resilience is likely to have the positive impact on employees’ perceptions about an organizational brand, and their cognitive and affective responses to it. Considering the findings of prior research, we hypothesize positive effects of organizational resilience on the core dimensions of internal brand equity.

#### H2

Organizational resilience has positive effects on (a) brand orientation, (b) internal brand knowledge, (c) internal brand involvement, and (d) internal brand commitment.

### Self-service technology in retailing

Organizations following the general trend of adopting and integrating technological innovations, such as SST in retailing, are driven not only by internal objectives, for example to create a seamless customer experience within a particular store, but also by the continuously evolving expectations of the society (Burke 2002; Weijters et al. 2007; Verhoef et al. 2009; Lin and Hsieh 2011; Reinartz et al. 2011). Prior research draws attention to the fact that the modern “working” consumer utilizing new technologies needs additional support, which is expected to be provided by retail employees and extend beyond fully taking over an operation (Cassidy et al. 2015; Collier et al. 2017). As a result, novel technological innovations intervening human-to-human interactions create new demands and put more pressure on employees working in the continuously changing retail environment. Following the assumptions of the socio-technical systems theory (Pasmore et al. 1982), earlier studies on SST suggest that a firm’s SST readiness is influenced by diverse stakeholder groups, including managers, employees, consumers, and channel members (Ramaseshan et al. 2015). Despite acknowledging a variety of perspectives, which need to be considered for a successful adoption and implementation of SST, previous studies predominantly apply a consumer perspective, while other perspectives, including an employee perspective, are not sufficiently explored (Di Pietro et al. 2014; Taillon and Huhmann 2019).

In this study, we adopt an employee perspective to investigate employees’ perceptions about organizational resilience, to assess their views about the internal brand, and to examine the effects triggered by SST in retailing. Prior branding research confirms the relevance of considering perceptions of employees by demonstrating that, despite the technological progress, consumers still tend to view employees as the ones, who “humanize” an organizational brand and facilitate emotional connections between the brand and its consumers (Morhart et al. 2009). A literature review demonstrates that employee attitudes and employee support serve as important value elements during the consumers’ interactions with SST (Vakulenko et al. 2018). A study exploring consumer roles in a self-service system shows that certain groups of consumers find it challenging to participate in the value co-creation process, which includes using SST, and, therefore, need assistance from employees (Åkesson and Edvardsson 2018). Previous retailing studies applying a consumer perspective provide additional empirical evidence about the critical role of employees by indicating that, in cases of SST failure, one recovery strategy preferred by consumers is to switch to employees for receiving an interpersonal assistance (Zhu et al. 2013). A study exploring discursive accounts of consumers’ shopping practices demonstrates that the core external factors influencing their in-store experiences include availability of employees and assistance provided by them (Bulmer et al. 2018). A study conducted in the fast fashion context indicates that consumers are valuing the improvements made by retailers in their services (for example, additional support provided by a competent salesperson) higher than the improvements in technology (Rese et al. 2019). Another study involving a panel of self-service checkout users confirms that employee presence in the SST area represents an important situational factor, which affects consumers’ attitudes toward SST (Collier et al. 2015). Similar findings are also reported in prior research conducted in other empirical settings. For example, by considering the attitude–intention model, a study performed in a banking industry confirms that clients’ attitudes toward employees affect their intentions to use SST (Curran et al. 2003). Finally, prior research conducted in the retailing context provides evidence about the presence of an interpersonal influence chain, which suggests that employees have a critical impact on consumers and consequently on store performance (Maxham et al. 2008; Lichtenstein et al. 2010).

The presence of high degrees of resilience in an organization determines the capabilities needed for “recognizing the inevitability of setbacks and thoroughly analyzing, coping with, and learning from them” (Vogus and Sutcliffe 2012, p. 723). The implementation of technological innovations in the form of SST in the retail sector can be perceived by employees as technological turbulence, which requires a certain degree of organizational resilience to effectively manage such changes (Schriber et al. 2019). Therefore, the resilience capabilities of organizations can be expected to have a critical impact on the formation of employees’ attitudes towards SST and consequently on their perceptions about SST. The findings reported in earlier studies demonstrate the criticality of resilience and preceding organizational mindfulness, when adopting new technologies within organizations (Nwankpa and Roumani 2014; Aanestad and Jensen 2016). Considering these insights, we propose a hypothesis about a positive effect of organizational resilience on employees’ perceptions about SST performance.

#### H3

Organizational resilience has a positive effect on perceptions about performance of SST.

Overall, technological innovations, such as SST, can be expected to have a critical impact on employees’ attitudes and behaviours. Extant branding research demonstrates that general perceptions of employees about their employers and their organizational identification, which are formed based on their attitudes and expressed through their behaviours, determine employees’ long-term commitment and support for achieving organizational goals (Mael and Ashforth 1992; Dutton et al. 1994; Lichtenstein et al. 2010; Porricelli et al. 2014). Employees’ perceptions of the new technology can also change from positive to negative. For instance, a study conducted in the hospitality industry indicates that in the initial stage of SST implementation, for example when technological innovations are introduced by managers, employees can feel enthusiasm, excitement, and engagement (Montargot and Ben Lahouel 2018). However, later when employees experience negative interferences in their work caused by SST, they can feel frustrated and anxious (Montargot and Ben Lahouel 2018). Prior research on SST suggests that perceptions about performance of SST can be used as a proxy for the general SST experience (Nijssen et al. 2016). Therefore, employees’ negative experiences with SST, which are characterized by negative perceptions about SST performance, can trigger their negative attitudes and behaviours. In contrast, positive perceptions about performance of SST can be expected to enhance a sense of psychological ownerships towards the employer’s brand that, based on evidence provided in previous studies, can facilitate positive brand-related attitudes and behaviours (Chang et al. 2012). Prior research on internal brand equity confirms these assumptions by highlighting the vital role of organizational context and various organizational factors in shaping employees’ responses to an organizational brand, which underlie the dimensions of internal brand equity (Boukis and Christodoulides 2020). Furthermore, previous studies indicate that a successful implementation of new technologies can empower employees and have positive effects on internal branding outcomes (Li et al. 2018). Therefore, positive perceptions about performance of SST can be hypothesized to have positive effects on cognitive and affective outcomes of internal brand management captured by the core dimensions of internal brand equity.

#### H4

Perceptions about performance of SST have positive effects on (a) brand orientation, (b) internal brand knowledge, (c) internal brand involvement, and (d) internal brand commitment.

**Fig. 1:**
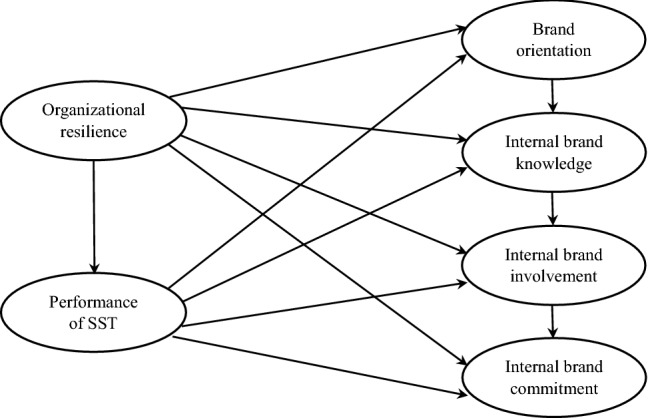
Conceptual model

The conceptual model demonstrates the hypothesized effects of organizational resilience and perceptions of employees about the performance of SST on the dimensions of internal brand equity (see Fig. 1).

## Methodology

The study was conducted by collecting data among employees of grocery stores in Sweden. The respondents, who were members of a panel of one large marketing research organization, were reached by distributing an online survey. The survey was distributed in two rounds. In the first round, the survey was sent to a profiled sample, which included employees of grocery stores. The data collection conducted by using a profiled sample resulted in a response rate of 44.01%. In the second round, the survey was sent to a general unprofiled sample of panel members. The data collection conducted by using an unprofiled sample resulted in a response rate of 28.04%. The introductory letter to the survey specified that the study focused on perceptions of employees working at grocery stores.

Overall, the data collection resulted in 270 valid responses. Following the procedure for assessing nonresponse error (Collier and Bienstock 2007), we compared the responses provided by the early respondents (*n* = 135) and the late respondents (*n* = 135). Since the independent samples *t* test did not identify any significant differences between these two groups across all variables, the findings were not affected by nonresponse error. Approximately matching the distribution of market shares in the Swedish grocery industry, the majority of the respondents (78.51%) were employees of the three largest grocery chains, which are ICA, Coop, and Axfood. These three market leading grocery chains, as well as their competitors, use a wide diversity of formats for their grocery stores ranging from a small convenience store to a large supermarket. In the sample, the majority of respondents were women (54.81%) and had more than 5 years of work experience in the grocery industry (62.96%). The age groups had the following distribution: less than 30 years old—35.92%, 30–49 years old—41.85%, and over 50 years old—22.22%.

The survey included scales measuring the four dimensions of internal brand equity: brand orientation, internal brand knowledge, internal brand involvement, and internal brand commitment. The scales were adapted from a previous study on internal brand equity (Baumgarth and Schmidt 2010). In addition, the survey included scales measuring organizational resilience and employees’ perceptions about performance of SST. The scales for these constructs were adapted from prior research on organizational resilience (Mu and Butler 2009) and SST (Nijssen et al. 2016). The items for each construct were measured on a 5-point Likert-type scale. Table 1 shows the descriptive statistics for each construct and correlation coefficients. The scale items used for measuring the constructs are presented in the “Appendix”.

**Table 1:** Descriptive statistics and correlations

Constructs	Mean (SD)	(1)	(2)	(3)	(4)	(5)	(6)
(1) Brand orientation	3.85 (0.88)	(0.73)	0.50	0.48	0.41	0.49	0.12
(2) Internal brand knowledge	3.90 (0.90)	0.71*	(0.70)	0.50	0.44	0.46	0.10
(3) Internal brand involvement	3.84 (0.85)	0.69*	0.71*	(0.66)	0.46	0.37	0.10
(4) Internal brand commitment	3.60 (0.90)	0.64*	0.66*	0.68*	(0.59)	0.35	0.13
(5) Organizational resilience	3.80 (0.86)	0.70*	0.68*	0.61*	0.59*	(0.62)	0.10
(6) Performance of SST	3.61 (0.95)	0.34*	0.32*	0.31*	0.36*	0.31*	(0.64)

Values on the diagonal—AVE (average variance extracted); values below the diagonal—correlations; values above the diagonal—squared correlations

*Correlation is significant at the 0.01 level (two-tailed)

## Analysis and results

We analysed the data by using IBM SPSS Statistics 27 and IBM SPSS Amos 27. In the initial stage of the analysis, we conducted a Harman’s single-factor test to assess the common method bias (Podsakoff et al. 2003). The results of the exploratory factor analysis based on the principal components method did not indicate a single predominant factor. The restriction of the extracted number of factors to one resulted in a solution, which extracted 49.51% of total variance. Considering the results of these tests, we did not identify a common method bias in this study.

The results of the confirmatory factor analysis are shown in Table 2. We conducted the confirmatory factor analysis by using the principal components method. The varimax rotation with Kaiser normalization converged in seven iterations. The solution including six extracted factors explained 78.20% of total variance. To assess reliability and validity, we calculated the Cronbach’s alpha coefficient, average variance extracted, and construct reliability for each construct. The results showed that all independent and dependent variables used in the analysis had acceptable levels of reliability and validity (see Table 3).

**Table 2:** Constructs and factor loadings

Constructs	Scale items	Factor 1	Factor 2	Factor 3	Factor 4	Factor 5	Factor 6
Brand orientation	BO1	0.71					
	BO2	0.78					
	BO3	0.72					
Internal brand knowledge	IBK1		0.72				
	IBK2		0.71				
	IBK3		0.72				
Internal brand involvement	IBI1			0.69			
	IBI2			0.84			
	IBI3			0.56			
Internal brand commitment	IBC1				0.61		
	IBC2				0.75		
	IBC3				0.79		
Organizational resilience	OR1					0.69	
	OR2					0.78	
	OR3					0.81	
	OR4					0.63	
Performance of SST	SST1						0.83
	SST2						0.86
	SST3						0.86

Principal component analysis, varimax rotation method with Kaiser normalization

**Table 3:** Constructs and standardized loadings

Constructs	Scale items	Standardized loadings*
Brand orientation (*α* = 0.89; AVE = 0.73; CR = 0.89)	BO1	0.85
	BO2	0.87
	BO3	0.85
Internal brand knowledge (*α* = 0.87; AVE = 0.70; CR = 0.87)	IBK1	0.75
	IBK2	0.89
	IBK3	0.86
Internal brand involvement (*α* = 0.86; AVE = 0.66; CR = 0.86)	IBI1	0.85
	IBI2	0.80
	IBI3	0.79
Internal brand commitment (*α* = 0.82; AVE = 0.59; CR = 0.81)	IBC1	0.82
	IBC2	0.76
	IBC3	0.73
Organizational resilience (*α* = 0.86; AVE = 0.62; CR = 0.87)	OR1	0.76
	OR2	0.77
	OR3	0.83
	OR4	0.78
Performance of SST (*α* = 0.84; AVE = 0.64; CR = 0.84)	SST1	0.78
	SST2	0.77
	SST3	0.85

α—Cronbach’s alpha, AVE—average variance extracted; CR—construct reliability

*p < 0.01

We used structural equation modelling for testing the proposed conceptual model. We applied the maximum likelihood estimation method. The structural model examined the effects between six variables including organizational resilience, performance of SST, and four dimensions of internal brand equity (i.e. brand orientation, internal brand knowledge, internal brand involvement, and internal brand commitment). Table 3 presents standardized loadings for each item across all constructs. For each construct included in the structural model, variance extracted and construct reliability showed sufficient levels (Hair et al. 2006). Table 4 demonstrates structural model estimates and fit indices. Based on the calculated fit indices, the structural model and the data had a good fit.

**Table 4:** Structural model estimates and fit indices

Hypothesis	Standardized estimates	Conclusion
H1a: Brand orientation → Internal brand knowledge	0.56 (*p* < 0.01)	Supported
H1b: Internal brand knowledge → Internal brand involvement	0.70 (*p* < 0.01)	Supported
H1c: Internal brand involvement → Internal brand commitment	0.61 (*p* < 0.01)	Supported
H2a: Organizational resilience → Brand orientation	0.76 (*p* < 0.01)	Supported
H2b: Organizational resilience → Internal brand knowledge	0.30 (*p* < 0.01)	Supported
H2c: Organizational resilience → Internal brand involvement	0.17 (*p* < 0.05)	Supported
H2d: Organizational resilience → Internal brand commitment	0.26 (*p* < 0.01)	Supported
H3: Organizational resilience → Performance of SST	0.36 (*p* < 0.01)	Supported
H4a: Performance of SST → Brand orientation	0.12 (*p* < 0.05)	Supported
H4b: Performance of SST → Internal brand knowledge	0.04 (*p* = 0.43)	No support
H4c: Performance of SST → Internal brand involvement	0.06 (*p* = 0.26)	No support
H4d: Performance of SST → Internal brand commitment	0.11 (*p* < 0.05)	Supported

Fit indices: *χ*^2^ = 333.15, *df* = 140, *χ*^2^/*df* = 2.38, RMSEA = 0.07, GFI = 0.89, AGFI = 0.84, NFI = 0.91, CFI = 0.94

The results demonstrated significant positive effects of brand orientation on internal brand knowledge (*β* = 0.56, *p* < 0.01), internal brand knowledge on internal brand involvement (*β* = 0.70, *p* < 0.01), and internal brand involvement on internal brand commitment (*β* = 0.61, *p* < 0.01). The findings supported hypotheses H1a, H1b, and H1c about the hierarchy of effects between the core dimensions of internal brand equity. Organizational resilience was found to have significant positive effects on brand orientation (*β* = 0.76, *p* < 0.01), internal brand knowledge (*β* = 0.30, *p* < 0.01), internal brand involvement (*β* = 0.17, *p* < 0.05), and internal brand commitment (*β* = 0.26, *p* < 0.01). As a result, hypotheses H2a, H2b, H2c, and H2d about the effects of organizational resilience on the dimensions of internal brand equity were supported. The findings confirmed hypothesis H3 about the positive effect of organizational resilience on employees’ perceptions about performance of SST (*β* = 0.36, *p* < 0.01). Employees’ perceptions about performance of SST were found to have significant positive effects on brand orientation (*β* = 0.12, *p* < 0.05) and internal brand commitment (*β* = 0.11, *p* < 0.05), confirming hypotheses H4a and H4d. The results did not indicate any significant direct effects of employees’ perceptions about performance of SST on internal brand knowledge (*β* = 0.04, *p* = 0.43) and internal brand involvement (*β* = 0.06, *p* = 0.26). Therefore, hypotheses H4b and H4c were not supported. In general, the findings demonstrated the importance of organizational resilience and employees’ perceptions about SST for enhancing the core dimensions of internal brand equity and, consequently, for building a strong internal brand.

## Discussion

The findings of this study provide evidence about the presence of hierarchical effects between the core dimensions of internal brand equity. The support for hypotheses H1a, H1b, and H1c demonstrates that internal brand equity emerges from brand orientation to internal brand knowledge, and then from internal brand involvement to internal brand commitment. The hierarchy of effects between the core dimensions of internal brand equity indicates that systematic efforts devoted within an organization to enhance brand orientation build a solid foundation for positive cognitive and affective responses of employees to internal branding. Furthermore, these responses influence the formation of internal brand commitment, which represents one impactful outcome of internal brand management. The results advance the assumptions proposed in prior research about possible effects between the dimensions of internal brand equity (Baumgarth and Schmidt 2010; Piehler et al. 2016; Boukis and Christodoulides 2020; Ngo et al. 2020). More specifically, the study validates the hierarchical nature of these effects and the evolving impact of internal branding on an organizational brand.

The study highlights that organizational resilience, which according to prior research can be expected to influence employees’ perceptions (Kuntz et al. 2016; Hartmann et al. 2020; Annarelli et al. 2020), represents a critical factor affecting internal brand equity. The confirmed hypotheses H2a, H2b, H2c, and H2d illustrate that organizational resilience has direct positive effects on brand orientation, internal brand knowledge, internal brand involvement, and internal brand commitment. Interestingly, organizational resilience has the strongest impact on brand orientation. Considering the strategic nature of brand orientation, this result indicates that organizational efforts devoted to increase organizational resilience are also beneficial for supporting the implementation of a strategic vision for an organizational brand and the enactment of brand values. The findings demonstrate that organizational resilience has an important role in influencing employees’ thoughts and emotions, which determine their commitment to the organizational brand.

Our findings confirm the notion that resilient organizations have capacities to effectively meet diverse challenges emerging in their day-to-day operations and mitigate negative influences arising in a workplace. The support for hypothesis H3 exemplifies how organizational resilience plays a major role in influencing opinions of employees about technological innovations implemented in their organizations. The study provides empirical evidence that resilience capabilities of organizations are critical for shaping employees’ perceptions of SST, which represent one type of technological innovations implemented in retailing. Furthermore, the support for hypothesis H4a demonstrates that these perceptions influence employees’ judgements about brand orientation. The significance of this effect can be explained by the notable role that technological innovations have in contemporary organizations (Burke 2002; Reinartz et al. 2011; Montargot and Ben Lahouel 2018). Therefore, the organizational efforts devoted to gaining a competitive advantage by adopting novel technologies become closely intertwined with implementing a strategic vision about the organizational brand. Nevertheless, considering the lack of support for hypotheses H4b and H4c, employees’ perceptions about performance of SST do not seem to possess sufficient strength for directly impacting cognitive and affective responses of employees to an organizational brand. One explanation can be that other organizational factors, for example psychosocial work environment, or specific internal branding activities, for example internal communication, might have a much stronger influence on shaping these responses than SST. Nevertheless, employees’ perceptions about SST can influence their internal brand commitment, as demonstrated by the support for hypothesis H4d, although this impact is much smaller compared to the direct effects of organizational resilience and internal brand involvement. Perhaps, these differences can be explained by the wide adoption of SST and its less controversial role than other forms of technologies, for example robotics and artificial intelligence with the potential to further threaten the job security of employees in the retail sector.

## Conclusions

The main purpose of this study was to investigate the impact of organizational resilience on internal brand equity considering the effects triggered by SST in retailing. The study advances the literature on internal brand equity by demonstrating a hierarchy of effects between the four dimensions of internal brand equity, which are brand orientation, internal brand knowledge, internal brand involvement, and internal brand commitment. Following the propositions about potential hierarchical effects between the dimensions of brand equity (Baumgarth and Schmidt 2010; Piehler et al. 2016; Boukis and Christodoulides 2020; Ngo et al. 2020), we show how these effects evolve across the dimensions and enhance internal brand equity. The study makes a theoretical contribution to research on internal branding by validating the relevance of organizational resilience for building a strong organizational brand and increasing internal brand equity. In addition to confirming the general importance of organizational resilience, which was indicated in prior organization studies (e.g. Youssef and Luthans 2007; King et al. 2016), our findings highlight the central role of organizational resilience in impacting the core outcome of internal branding, which is internal brand equity. More specifically, the findings specify that organizational resilience has direct positive effects on all four dimensions of internal brand equity.

In this study, we provide further evidence about the criticality of developing resilience capabilities of organizations, which are important not only in contexts of extreme environments, but also in stable environments involving day-to-day operations. By considering a trigger in the form of SST, we examine its potential to mediate the impact of organizational resilience on internal brand equity. Building on the assumptions that organizational resilience can impact perceptions of employees about technological innovations implemented in their workplaces (Nwankpa and Roumani 2014; Aanestad and Jensen 2016), the study demonstrates the extent to which organizational resilience influences employees’ perceptions about performance of SST in retailing. Furthermore, the findings show that these perceptions have direct positive effects on brand orientation and internal brand commitment, as well as indirect positive effects on internal brand knowledge and internal brand involvement. By adopting an employee perspective for exploring the impact of technological innovations on internal branding, the study identifies unique organizational factors of central importance for achieving success in a technologically advanced marketplace.

## Managerial implications

The study provides practical recommendations for marketing managers responsible for internal branding of organizations. They are advised to systematically assess not only the achieved outcomes of internal branding, but also the changes in organizational resilience. Marketing managers are recommended to integrate issues related to resilience in the training of employees, because, as demonstrated by the findings of this study, organizational resilience can reinforce or undermine the outcomes of internal branding. To increase organizational resilience, training programmes can include resilience activities focusing on individuals, groups, as well as the entire organization, for example personal mindfulness practices, team building exercises, and organization-wide scenario-based trainings. In collaboration with HR specialists or organizational psychologists, marketing managers can plan and implement interventions needed for stimulating organizational resilience within their organizations. By working proactively with enhancing resilience, marketing managers can prepare employees not only for handling adversity and negative effects of using new technologies such as SST, but also for performing their ordinary work tasks more effectively. In general, marketing managers are encouraged to complement internal brand building activities with additional actions promoting possibilities for increasing organizational resilience.

Marketing managers are recommended to consider the fact that organizational resilience is also critical for supporting the adoption of technological innovations. While resilient organizations have capacities to mitigate potential negative effects, which can arise as a result of using new technologies, organizations lacking resilience capabilities might find it problematic to address such challenges. Consequently, negative perceptions of employees about performance of technological innovations, such as SST in retailing, can weaken the impact of internal brand management and even damage the organizational brand. Therefore, marketing managers are advised to follow closely changes in employees’ perceptions about technologies implemented at the workplace and monitor their impact on employees’ judgements about the organizational brand and the outcomes of internal branding. The data about the estimated impact of organizational resilience and employees’ perceptions about technological innovations can serve as an important complementary information in the managerial decision-making process.

The core dimensions of internal brand equity represent important factors, which should be considered in assessing the overall success of internal brand management. In their strategic plans, marketing managers are recommended to include clear and specific objectives addressing each dimension of internal brand equity, as well as to develop actions for enhancing each respective dimension. The presence of hierarchical effects between brand orientation, internal brand knowledge, internal brand involvement, and internal brand commitment indicates that marketing managers need to adopt a holistic view upon internal brand equity and to ensure that internal branding initiatives would address all of these dimensions. The core dimensions investigated in this study are central for capturing internal brand equity. Marketing managers can complement this approach to managing internal brand equity by considering additional aspects, which might be critical for assessing employees’ perceptions about their organizational brands and estimating their responses to internal branding.

## Limitations and future research

One limitation of this study is related to its empirical context, namely the retail sector in Sweden. Considering the increasing efforts of Swedish retailers in implementing technological innovations, this setting was beneficial for investigating perceptions about SST, resilience, and internal branding. Nevertheless, for future research, we recommend to explore these topics by conducting a cross-cultural study, which would enable a comparison of perceptual differences across various countries. Possible avenues for future research can also be to investigate diverse topics emerging from the combination of research streams on internal branding, resilience, and technological innovations, in other empirical settings, besides retailing, for example in different business-to-business markets or in the public sector. While the employee perspective adopted in this study was beneficial for advancing the current state of research, which mostly relies on a consumer perspective, it is still limited to just one internal stakeholder group. We suggest that future studies would apply a stakeholder perspective and consider multiple stakeholders influencing or being influenced by the implementation of technological innovations. Future research can assess the differences in perceptions about specific types of SST across various stakeholder groups, as well as consider other technologies, for example artificial intelligence, impacting the decision-making processes of multiple stakeholders. This study confirms the need to conduct future branding research exploring various organizational factors and to examine how these factors influence the outcomes of internal branding and organizational performance.

This study was initiated prior to the COVID-19 pandemic, and because of that we have no data on how the pandemic impacted organizational resilience and employees’ perceptions in the retail sector. This group of employees has provided important services to society, while they have received little if any recognition in media for their outstanding contributions. In short, they went from working in an ordinary environment to one that the authorities recommended that people should avoid. Consequently, it is of interest to examine the factors which affected organizational resilience and perceptions of retail employees during the pandemic. Furthermore, increased online shopping of groceries during the pandemic has further pushed consumers towards using SST resulting in a demand for extended services. An avenue for future research is, therefore, to examine how internal brand management in the retail sector can cope with and support the increased digitalization that these services include. Overall, the post-pandemic recovery represents an important and interesting setting for investigating organizational resilience, technological innovations, and internal branding.

## Appendix

See Table 5.

**Table 5:** Constructs and scale items

Constructs	Scale items
Brand orientation*	BO1. In our store, we have a clear idea of what our brand stands for
	BO2. We see our brand as a valuable asset
	BO3. The majority of employees in our store understand our brand values
Internal brand knowledge*	IBK1. I know how our brand is communicated (for example through newspapers, the Internet, etc.)
	IBK2. I am aware of the goals we try to achieve through our brand
	IBK3. I am well informed about the values that our brand stands for
Internal brand involvement*	IBI1. I am aware that our brand contributes significantly to the success of our store
	IBI2. I know that our brand is a decisive factor for the loyalty of our customers
	IBI3. I am convinced that our customers recommend our brand to others
Internal brand commitment*	IBC1. I am proud to tell others that I work for the grocery chain that owns this brand
	IBC2. My loyalty to the brand is based on the similarity between my values and the values that the brand has
	IBC3. The values that our brand stands for affect my daily behaviour
Organizational resilience*	OR1. In our store, we are committed to solving any problem that arises
	OR2. In case of a problem, we as employees can rely on each other
	OR3. We as employees do not give up until we have solved a problem
	OR4. In our store, we work actively to develop our skills and knowledge in managing problems
Performance of SST	How would you evaluate self-service technology used in the grocery chain that you work for?
	SST1. 1—Highly unsatisfying … 5—Highly satisfying
	SST2. 1—Time-consuming … 5—Time-saving
	SST3. 1—Not appealing … 5—Very appealing

*Scale: 1—strongly disagree…5—strongly agree
